# Evaluation of 18F-DCFPyL PSMA PET/CT for Prostate Cancer: A Meta-Analysis

**DOI:** 10.3389/fonc.2020.597422

**Published:** 2021-02-17

**Authors:** Ke-Hao Pan, Jin-Feng Wang, Chun-Ying Wang, Abdul Aziz Nikzad, Fang Q. Kong, Li Jian, Yin-Qiu Zhang, Xiao-Ming Lu, Bin Xu, Ya-Li Wang, Ming Chen

**Affiliations:** ^1^ Department of Urology, Affiliated Zhongda Hospital of Southeast University, Southeast University, Nanjing, China; ^2^ Lishui District People’s Hospital, Nanjing, China; ^3^ Department of Urology, Yancheng Third People’s Hospital, Yancheng, China; ^4^ Department of Urology, Affiliated Zhongda Hospital of Southeast University, Nanjing, China; ^5^ Department of Nosocomial Infection, Affiliated Zhongda Hospital of Southeast University, Nanjing, China; ^6^ Department of Urology, Jinhu People’s Hospital, Jinghua, China; ^7^ Department of Digestion, Affiliated Zhongda Hospital of Southeast University, Southeast University, Nanjing, China

**Keywords:** prostate cancer, 18F-DCFPyL PSMA PET/CT, diagnosis, meta-analysis, imaging

## Abstract

**Background:**

To systematically review the clinical value of 18F-DCFPyL prostate-specific membrane antigen positron emission tomography/computed tomography (PSMA PET/CT) in the diagnosis of prostate cancer (PCa).

**Methods:**

Literature concerning 18F-DCFPyL PSMA PET/CT in the diagnosis of prostate cancer published from 2015 to 2020 was electronically searched in the databases including PubMed and Embase. Statistical analysis was carried out with STATA 15 software, and the quality of included studies was tested with quality assessment of diagnostic accuracy studies (QUADAS) items. The heterogeneity of the included data was tested.

**Results:**

In total, nine pieces of literature involving 426 patients met the inclusion criteria. The heterogeneity of the study group was not obvious. The SEN, SPE, LR+, LR−, DOR as well as AUC of 18F-DCFPyL PSMA PET/CT diagnosis of prostate cancer were 0.91, 0.90, 8.9, 0.10, 93, and 0.93. The pooled DR of 18F-DCFPyL labeled PSMA PET/CT in PCa was 92%. The pooled DR was 89% for PSA≥0.5 ng/ml and 49% for PSA < 0.5ng/ml.

**Conclusion:**

18F-DCFPyL PSMA PET/CT had good sensitivity and specificity for the diagnosis of prostate cancer. The DR of 18F-DCFPyL PSMA PET/CT was correlated with PSA value. Further large-sample, high-quality studies were needed.

## Introduction

Prostate cancer is the most common tumor of the male genitourinary system. In recent years, the number of patients with prostate cancer in China had been increasing (1). Epidemiological studies had found that the incidence of prostate cancer in men in China had risen significantly in the past two decades. The popularity of health examinations had increased the detection rate of prostate cancer patients. In particular, the rapid development of imaging technology significantly improved the detection rate of early PCa (2). Prostate-specific membrane antigen (PSMA) was highly expressed in prostate cancer cells and was up-regulated in poorly differentiated, advanced, metastatic, and hormone-independent prostate cancer cells. Its positive detection rate in prostate cancer was higher than that of PSA. It had become a diagnostic tool for prostate cancer (3). 18F-DCFPyL was a kind of 18F-labeled PSMA, which had high imaging quality and diagnostic value.

The high incidence and fatality rate of prostate cancer seriously threatened men’s life and health (4, 5). PSMA was expressed on the surface of normal prostate and prostate hyperplasia cells and was significantly up-regulated in most prostate cancer cells. It was a specific molecular marker for prostate cancer. Radionuclide-labeled PSMA tiny molecule inhibitors had shown great clinical application value in prostate cancer detection and treatment evaluation. Based on the pharmacodynamic group Glu-urea-Lys, 68Ga-PSMA-11 was the first glutamate-urea small molecule PET imaging agent with good biological distribution characteristics (6–9). However, the nuclide 68Ga was obtained by the generator and had a short half-life. The 68Ga had high positive electron energy, which made the signal-to-noise ratio of PET images low, and its clinical application was limited. 18F was the most widely used positron nuclide in clinical practice. 18F-DCFPyL was also a PSMA specific small molecule imaging agent developed based on the Glu-urea-Lys structure. It had the characteristics of high affinity and good pharmacokinetics *in vivo*. The performance was better than 68Ga-PSMA-11 (10–12).

To further explore the accuracy and reliability of 18F-DCFPyL PSMA PET/CT in prostate cancer diagnosis, this study collected relevant literature and conducted a meta-analysis of its data summary.

## Methods

### Search Strategy

Computer searches included PubMed, Embase to collect relevant literature on prostate cancer diagnosis by 18F-DCFPyL PSMA PET/CT. The search period was from January 2015 to 2020 October. Subject terms included 18F-DCFPyL PSMA PET/CT, prostate, prostate cancer, and prostate tumor, and the search method was adjusted according to the specific database. The search strategy was determined after multiple pre-searches. Using a combination of database retrieval and manual retrieval, two evaluators independently retrieved and researched the included literature’s references. No language restrictions were used.

### Exclusion Criteria

Repeated publications; research results could not be extracted or transformed into data required for analysis; Case, Case reports, reviews; Patients with a history of other tumors.

### Literature Screening

Literature was independently screened by two reviewers based on the inclusion criteria, first reading the title and abstract. After cross-checking the results, data were extracted from cohort studies. The essential/primary characteristics of the included literature (11, 13–20) were shown in **Table 1**. The four grid table data and detection rate were shown in **Table 2**. The study field of prostate cancer of the included studies was shown in **Table 3**.

**Table 1:** Study and patient characteristics.

Studies	Year	Size	Mean Age	Country	Study type	PSA (ng/ml)	Gleason Score
Liu Yachao (13)	2020	49	66.3	China	Retrospective	1.2	≤6: 5% 7: 50% ≥8: 34% unknown: 11%
Rousseau (14)	2019	130	69.1	Canada	Prospective	5.2	≤6: 13% 7: 50% ≥8: 37%
Wondergem (15)	2017	34	67.8	Netherlands	Retrospective	0.6	≤6: 8% 7: 50% ≥8: 42%
Dietlein (16)	2017	62	70	Germany	Retrospective	3.2	≤6: 7% 7: 56% ≥8: 37%
Dietlein (17)	2015	14	68	Germany	Retrospective	4.4	≤6: 8% 7: 50% ≥8: 42%
Song (18)	2020	72	71.5	USA	Prospective	3.0	≤6: 8% 7: 51% ≥8: 40%
Rowe (19)	2020	31	63	UK	Prospective	0.4	≤2: 26% 3: 42% ≥4: 32%
Gorin (20)	2018	25	61	UK	Prospective	9.3	7: 4% 8: 44% 9: 52%
Rowe (11)	2016	9	71	UK	Prospective	8.8	7: 44% 9: 44% 10: 12%

**Table 2:** The four grid table and the detection rate of the included studies.

Authors	TP	FP	FN	TN	Overall DR	DR in Patients with PSA < 0.5	DR in Patients with PSA ≥ 0.5
Liu Yachao (13)	90	12	10	87	47/49(95.9%)	8/10(80%)	38/39(97.4%)
Rousseau (14)	96	11	4	89	110/130 (84.6%)	3/5 (60%)	107/125 (85.6%)
Wondergem (15)	92	5	8	89	28/34 (77.8%)	2/5(40%)	26/29(90.0%)
Dietlein (16)	95	15	5	85	46/62 (74.2%)	1/8 (12.5%)	45/54 (83.3%)
Dietlein (17)	91	2	7	88	12/14(85.7%)	2/4 (50%)	10/10(100%)
Song (18)	90	15	10	85	61/72(84.7%)	4/8(50%)	57/64(89.1%)
Rowe (19)	89	12	11	88	24/31(77.4&)	5/10(50%)	19/21(90.1%%)
Gorin (20)	90	12	10	88	/	/	/
Rowe (11)	92	10	8	90	129/135(95.5%)	4/9(44.4%)	115/126(91.6%)
Pooled values					92%	49%	89%

**Table 3:** The study field of prostate cancer of the included studies.

Studies	Year	PCa
Liu Yachao (13)	2020	Preoperative diagnosis
Rousseau (14)	2019	BCR
Wondergem (15)	2017	BCR
Dietlein (16)	2017	BCR
Dietlein (17)	2015	BCR
Song (18)	2020	BCR
Rowe (19)	2020	BCR
Gorin (20)	2018	Preoperative diagnosis
Rowe (11)	2016	Widespread cancer metastasis

### Quality Assessment

Two data extractors used the QUADAS quality evaluation tool to evaluate the quality of the included literature according to “yes” (satisfying the standard), “no” (not meeting the standard), and “unclear” (unavailable information). If there was a dispute, both parties discussed and reached an agreement (4).

### Statistical Analysis

The data of the four grid table extracted from the included literature was sorted and statistically analyzed. The Q test was used for heterogeneity, and its I^2^ value was calculated. If I^2^ ≤50%, it indicated low heterogeneity. The combined sensitivity, combined specificity, diagnostic odds ratio (DOR), positive likelihood ratio (LR+) and negative likelihood ratio (LR−) of the included literature were written, and the receiver operating characteristic curve (SROC) was drawn. The area under the curve (AUC) was calculated. To detect the literature’s bias, STATA 15 software was used to draw the Deeks funnel chart and calculate the corresponding P value.

## Results

### Features of Included Literature

**Figure 1:**
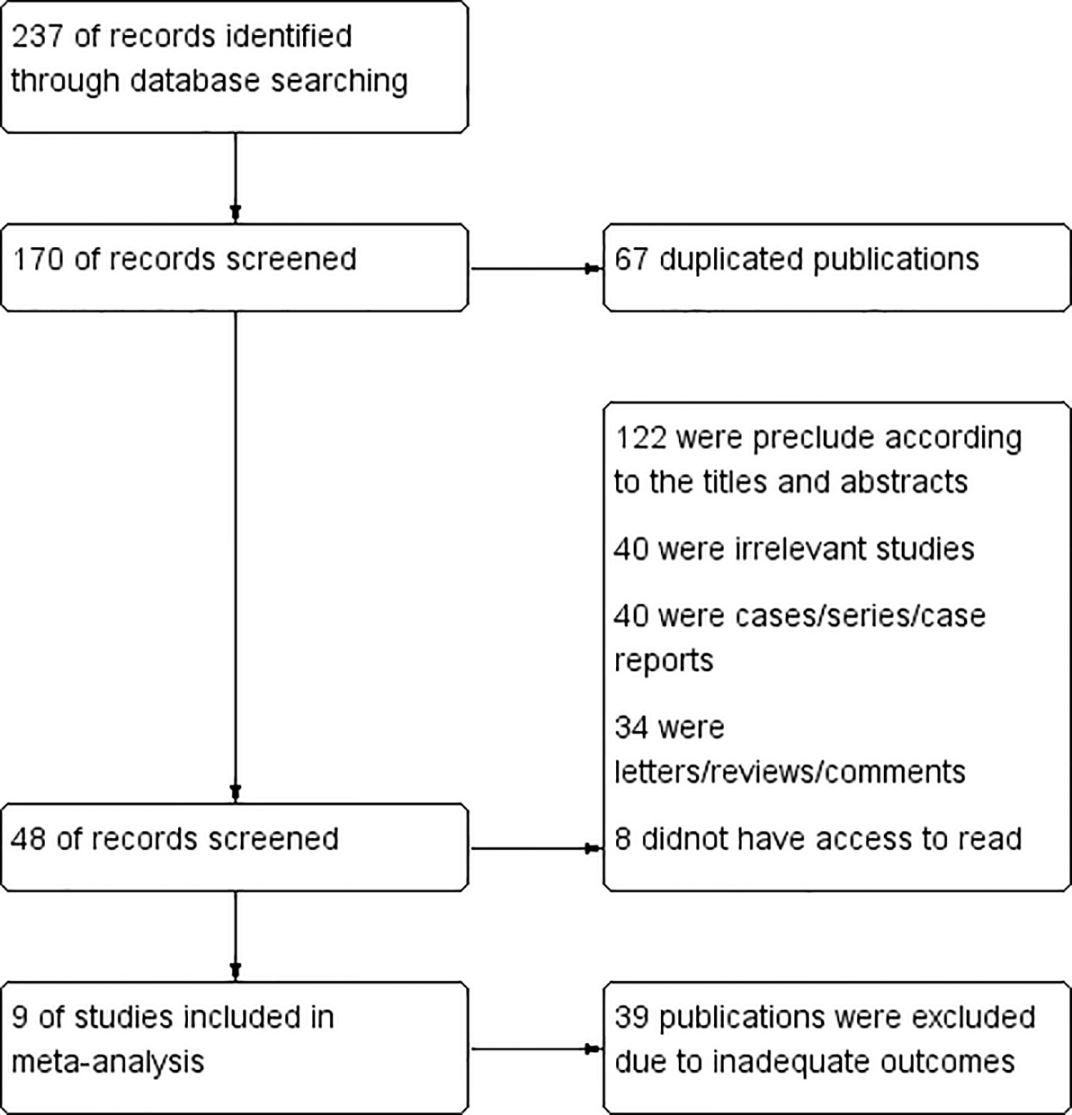
Flow Chart.

A total of 237 related articles were initially obtained after searching the corresponding database based on keywords. After excluding duplicates and reading the abstract, nine related articles were obtained (11, 13–20). The characteristics of the studies were illustrated in **Table 1**. The flow chart was shown in **Figure 1**. In total, nine literature involving 426 patients met the inclusion criteria. Seven articles studied the BCR of PCa. One study was conducted on the preoperative diagnosis of PCa, and the other one studied the widespread metastasis of PCa.

### Qualitative Analysis

**Figure 2:**
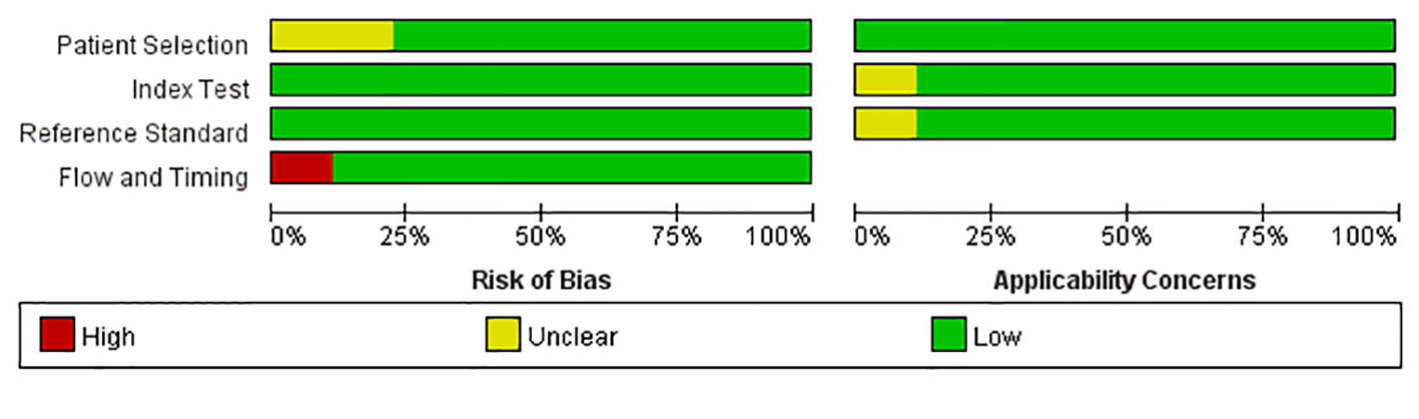
Quality evaluation of the inccluded studies.

The literature QUADAS scale displayed that the included articles were of high quality. The research quality evaluation was shown in **Figure 2**.

### Detection Rate

The DR of 18F-DCFPyL PET/CT was shown in **Table 2**. The pooled detection rate (DR) of 18F-DCFPyL PSMA PET/CT in PCa was 92%. The pooled DR was 89% for PSA ≥0.5 ng/ml and 49% for PSA <0.5 ng/ml. The DR of 18F-DCFPyL PSMA PET/CT was correlated with the PSA value. The higher the PSA value was, the higher the DR of 18F-DCFPyL PSMA PET/CT was.

### Meta-Analysis

**Figure 3:**
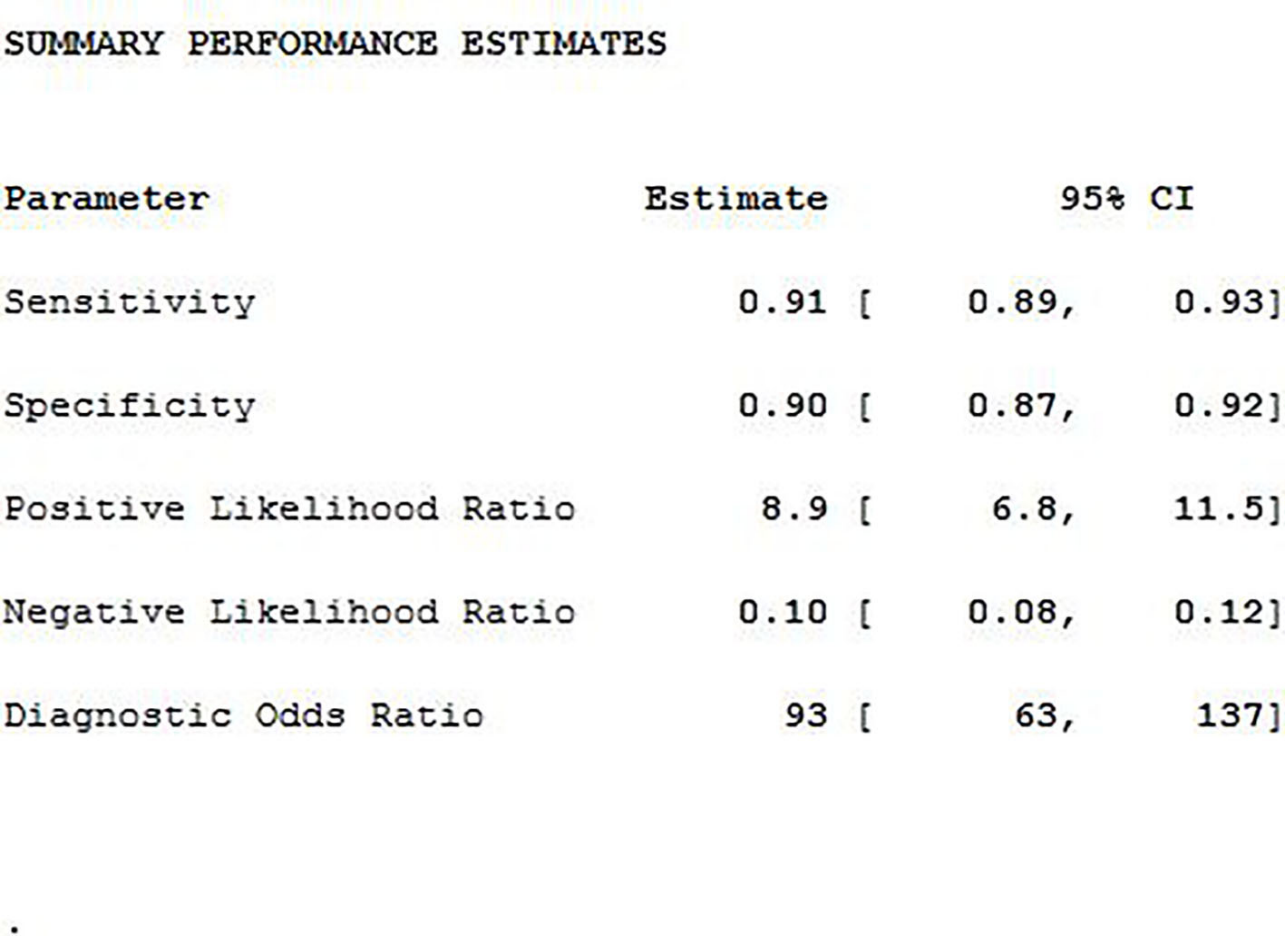
The combined statistics.

**Figure 4:**
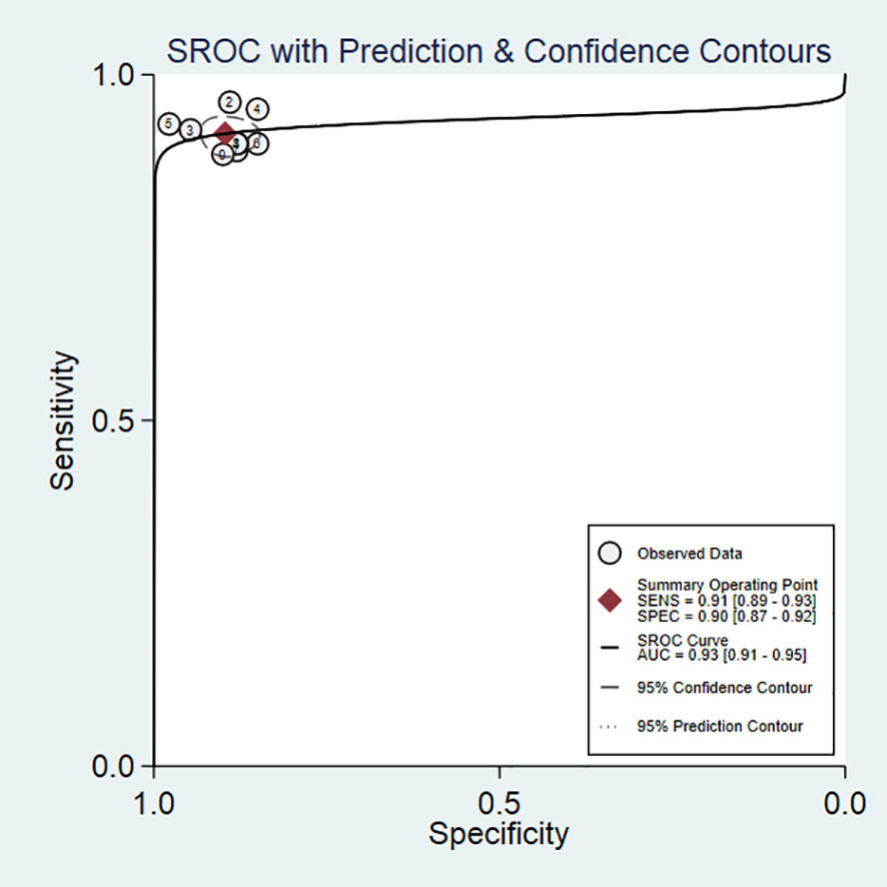
SROC curves of 18F-DCFPyL PSMA PET/CT.

**Figure 5:**
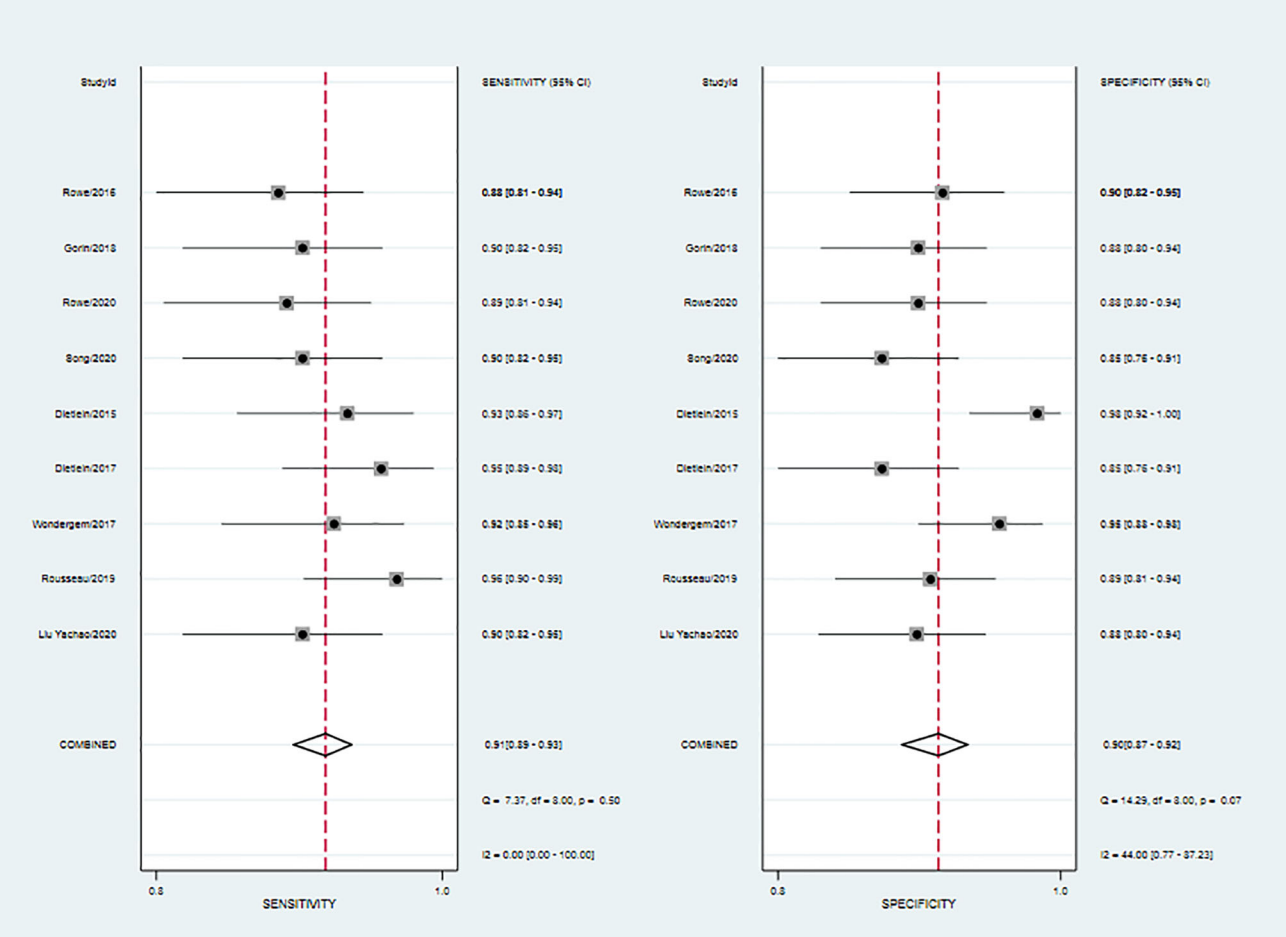
Forest map of 18F-DCFPyL PSMA PET/CT.

The SEN, SPE, LR+, LR−, DOR as well as AUC of 18F-DCFPyL PSMA PET/CT diagnosis of prostate cancer were 0.91, 0.90, 8.9, 0.10, 93, and 0.93. The results of the meta-analysis were presented in **Figure 3**. The SROC curve and the forest map of 18F-FACBC PET/CT were presented in **Figures 4** and **5**, respectively.

### Heterogeneity Analysis

As shown in **Figure 5**, 18F-DCFPyL PSMA PET/CT had no heterogeneity in the sensitivity of prostate cancer diagnosis (Q value, P-value, I^2^ value were 7.37, 0.50, 0.00, respectively). A random-effects model was used. The Spearman correlation coefficients of the sensitivity logarithm and (1-specificity) logarithm of 18F-DCFPyL PSMA PET/CT diagnosis of prostate cancer were −0.140 (P > 0.05), indicating that there was no threshold effect.

### Sensitivity Analysis

**Figure 6:**
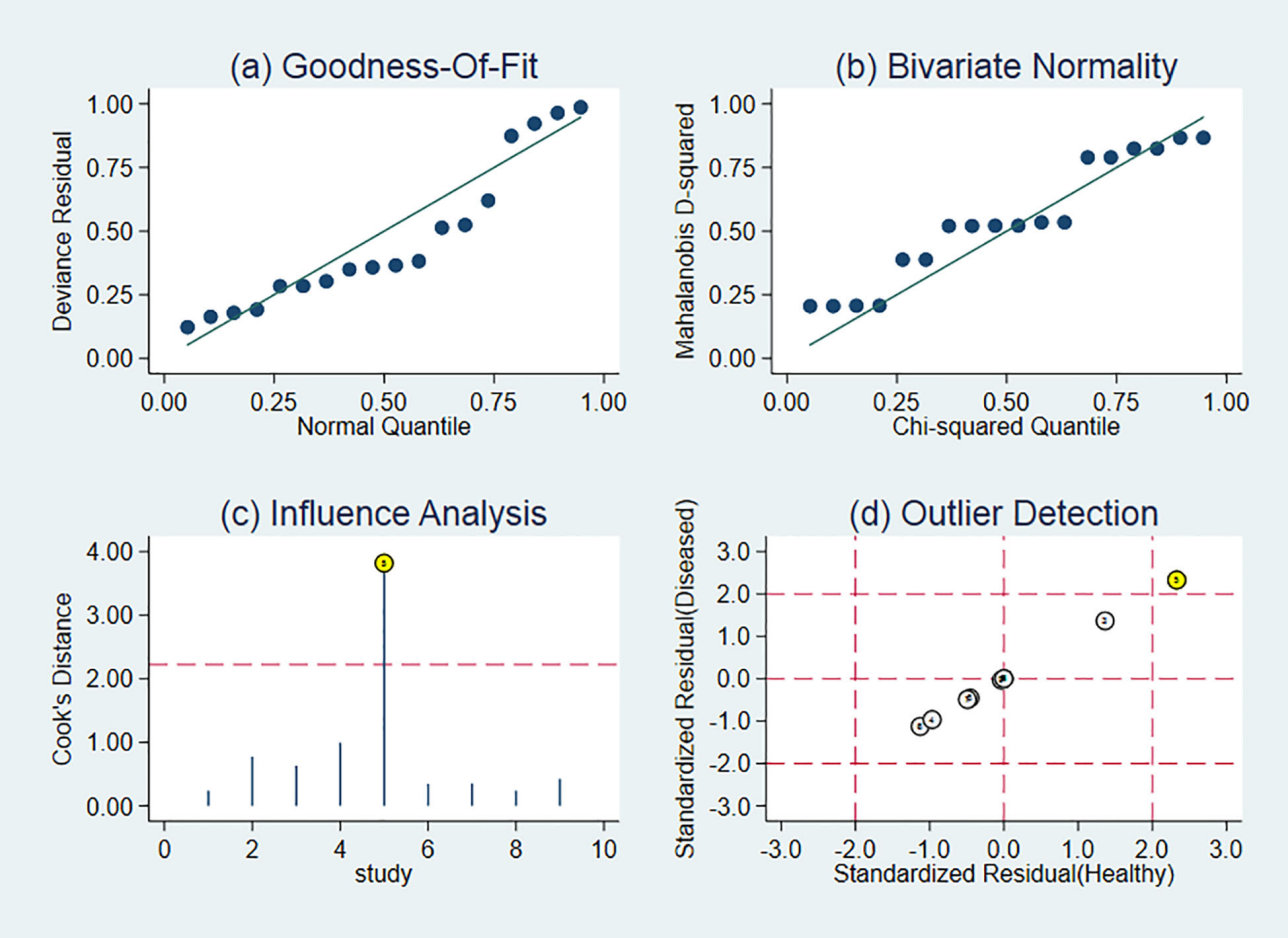
Sensitivity analysis of studies. Goodness of fit refers to the fitting degree of the regression line to the observed value. The statistic to measure goodness of fit is R^2^. The maximum value of R^2^ is 1. The closer the value of R^2^ is to 1, the better the fitting degree of the regression line to the observed value is; on the contrary, the smaller the value of R^2^ is, the worse the fitting degree of the regression line to the observed value is.

**Figure 6** revealed a sensitivity analysis. Each selected literature was excluded one by one, and its combined sensitivity, combined specificity, and DOR were recalculated. Compared with the exclusion results, there was no significant change, indicating that the results of the included literature were highly reliable.

### Clinical Analysis

**Figure 7:**
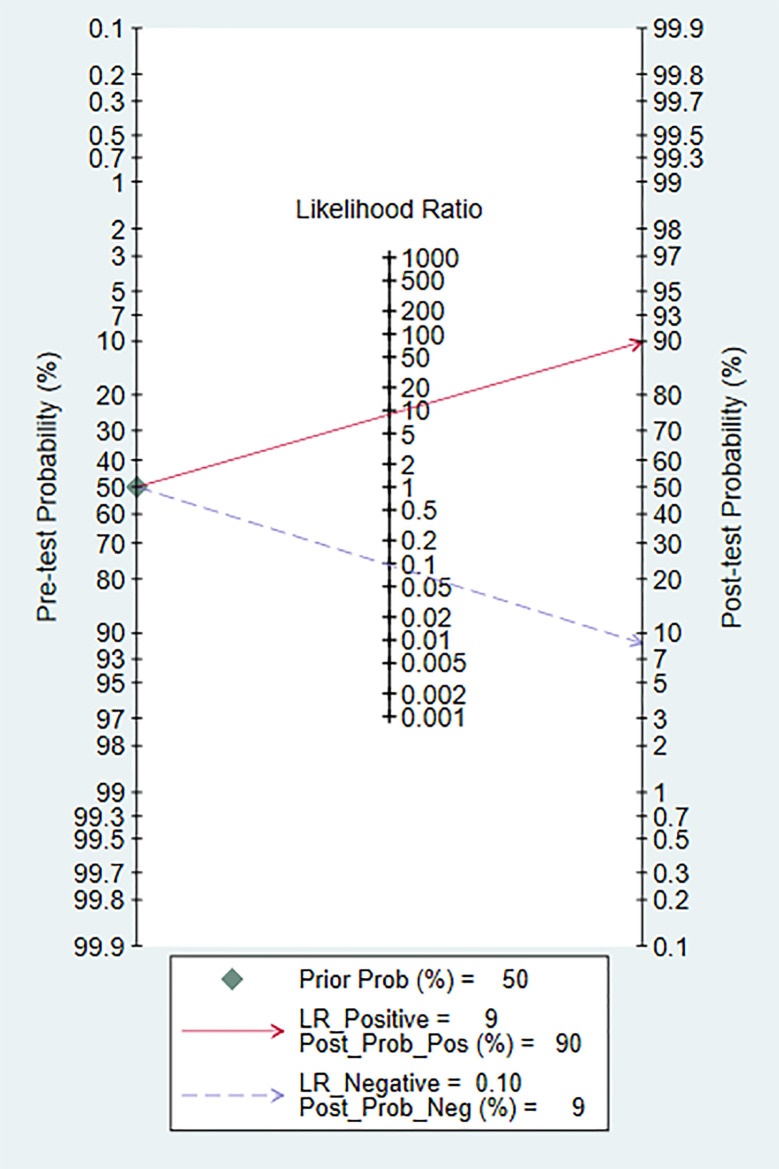
The Fagan map.

The Fagan diagram was constructed for clinical analysis. It was shown in **Figure 7**. The post-test probability of 18F-DCFPyL PSMA PET/CT was 90%, which was higher than the pre-test probability (50%).

### Publication Bias

**Figure 8:**
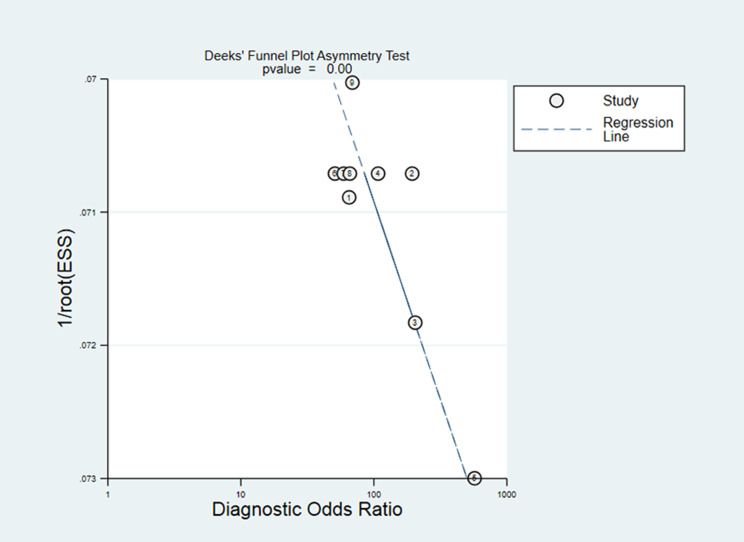
Deeks’ test.

The drawn Deeks funnel chart suggested that 18FDCFPyL PET/CT had publication bias in diagnosing prostate cancer (p = 0.00). **Figure 8** illustrated the Deeks funnel chart. However, the sensitivity analysis showed that our results are stable. Despite publication bias, our sensitivity test found that the article is stable, indicating that our results are reliable.

## Discussion

PCa is one of the most common malignant tumors in men. However, the biological behavior and clinical conditions of PCa have significant heterogeneity, which makes it very difficult to diagnose. As a result, most patients miss the best treatment time. Therefore, imaging technology with high sensitivity and accuracy is incredibly crucial. There are many imaging evaluation methods for PCa, including CT, MRI, and ultrasound. Traditional imaging methods, such as CT or MRI, have low accuracy identifying metastatic lesions and have low diagnostic efficiency for early lymph node metastasis and distant metastasis. PET/CT technology combines the unique advantages of anatomy image and functional metabolism image so that doctors can obtain accurate anatomical positioning while understanding the biological metabolism information (21). Therefore, this method has important clinical significance for the diagnosis of PCa. With the development of PET/CT imaging agents, more and more radioactive imaging agents are used, such as 18F-FDG, 11C-choline, 18F-DCFPyL, 68Ga-PSMA, *etc*. The use of these different imaging agents further improves the sensitivity and specificity of PET/CT in the diagnosis of PCa (22).

The expression of PSMA corresponds to the grade and stage of cancer at the histopathological level, promoting the development of many radiopharmaceuticals targeting PSMA. PSMA targeted PET imaging has significant sensitivity and specificity in detecting PCA sites in various disease states. Based on the pharmacodynamic group Glu-urea-Lys, 68Ga-PSMA was the first glutamate-urea small molecule PET imaging agent with good biological distribution characteristics.

There are multiple meta-analyses of 68Ga-PSMA. Perera found that the summary sensitivity and specificity of 68Ga-PSMA in PCa were both 86% (23). Kimura found that the pooled sensitivity using lesion-based and field-based analyses were 0.84 and 0.82, respectively (24). However, the nuclide 68Ga was obtained by the generator and had a short half-life. The 68Ga had high positive electron energy, which made the signal-to-noise ratio of PET images low, and its clinical application was limited. 18F-DCFPyL is a PSMA-targeted PET agent that may be nearing regulatory approval in the US and has been increasingly used in many centers worldwide. It had the characteristics of high affinity and good pharmacokinetics *in vivo*. The performance was better than 68Ga-PSMA-11. Markowski found that long prostate-specific antigen doubling times were associated with pelvic confined prostate cancer (25, 26).

This study’s results demonstrated that the weighted sensitivity, specificity, positive likelihood ratio, negative likelihood ratio, diagnostic odds ratio were 0.91, 0.90, 8.9, 0.10, and 93, respectively, and the area under the curve was 0.93. This suggested that 18F-DCFPyL could be used as a diagnostic tool for prostate cancer. The pooled detection rate (DR) of 18F-DCFPyL PSMA PET/CT in Pca was 92%. The pooled DR was 89% for PSA ≥0.5 ng/ml and 49% for PSA <0.5 ng/ml. The DR of 18F-DCFPyL PSMA PET/CT was correlated with the PSA value. The higher the PSA value was, the higher the DR of 18F-DCFPyL PSMA PET/CT. Therefore, for prostate cancer patients, strengthening PSA testing combined with 18F-DCFPyL PSMA PET/CT could improve the diagnostic level. Our study shows that 18F-DCFPyL PSMA PET/CT is excellent in preoperative diagnosis, biochemical recurrence, and metastasis.

This research still had some limitations. First, the number of documents was limited, and retrospective studies accounted for a large amount, so that could cause selection bias. Second, due to the literature’s different publication time, the 18F-DCFPyL PET/CT diagnostic standards in some literature had specific differences, which could affect the results. Third, there was poor-quality research in these documents, which led to article’s publication bias.

In summary, the 18F-DCFPyL PET/CT method had high efficiency in diagnosing prostate cancer. CT and MRI were useful non-invasive methods for diagnosing prostate cancer. However, the meta- analysis of the value of CT and MRI in diagnosing prostate cancer displayed that the sensitivity and specificity of CT are 42 and 82%, respectively. The sensitivity and specificity of MRI were 39 and 82%, respectively. Hence, 18F-DCFPyL PET/CT could become the first choice for prostate cancer in the near future.

## Conclusion

This study showed that 18F-DCFPyL PET/CT had high sensitivity and specificity for the diagnosis of prostate cancer. At present, with the popularization of PET/CT in the clinic, it will provide further imaging basis for the diagnosis and treatment of prostate cancer and have essential clinical value for improving the survival rate of patients with prostate cancer.

## Data Availability Statement

The raw data supporting the conclusions of this article will be made available by the authors, without undue reservation.

## Author Contributions

K-HP, J-FW, C-YW contributed equally to this work and should be considered co-first authors. X-ML, BX, Y-LW and MC contributed equally to this work and should be considered the co-corresponding authors. AAN, FQK, LJ and Y-QZ contributed equally to this work and should be considered co-second author.

## Funding

This study was funded by The National Natural Science Foundation of China (No. 81872089, 81370849, 81672551, 81300472, 81070592, 81202268, 81202034), Natural Science Foundation of Jiangsu Province (BK20161434, BL2013032, BK20150642 and BK2012336), Six talent peaks project in Jiangsu Province, Jiangsu Provincial Medical Innovation Team (CXTDA2017025), The National Key Research and Development Program of China (SQ2017YFSF090096), Jiangsu Provincial Key Research and Development Program (BE2019751), Innovative Team of Jiangsu Provincial (2017ZXKJQWO7), Jiangsu Provincial Medical Talent (ZDRCA2016080).

## Conflict of Interest

The authors declare that the research was conducted in the absence of any commercial or financial relationships that could be construed as a potential conflict of interest.

## Abbreviations

18F-DCFPyL, (2-(3-{1-carboxy-5-[(6-18F-flfluoro-pyridine-3-carbonyl)-amino]-pentyl}-ureido)-pentanedioic acid); CT, X-ray computerized tomography; MRI, magnetic response imaging; PCa, prostate cancer; PET, positron emission tomography; PSA, prostate specific antigen; PSMA, prostate specific membrane antigen; SEN, sensitivity; SPE, specificity; SROC, summary receiver operating characteristic; DR, detection rate; TP, true positive; FP, false positive; FN, false negative; TN, true negative; QUADAS, Quality Assessment of Diagnostic Accuracy Studies; LR+, positive likelihood ratio; LR−, negative likelihood ratio; DOR, diagnostic value; AUC, area under curve; BCR, biochemical recurrence.
